# Exploring the implementation and theory of a school-based diet and active lifestyle intervention among primary school children

**DOI:** 10.3389/fpubh.2025.1708767

**Published:** 2025-12-15

**Authors:** Cindy Mei Jun Chan, Falk Müller-Riemenschneider, Michael Yong Hwa Chia, Mary Foong-Fong Chong, Zoe Jane-Lara Hildon

**Affiliations:** 1 Saw Swee Hock School of Public Health, National University of Singapore and National University Health System, Singapore, Singapore; 2 Center for Digital Health, Berlin Institute of Health (BIH), Charité-Universitätsmedizin Berlin, Berlin, Germany; 3 Physical Education and Sports Science Academic Department, National Institute of Education, Nanyang Technological University, Singapore, Singapore; 4 Institute for Human Development and Potential, Agency for Science, Technology and Research, Singapore, Singapore

**Keywords:** healthy eating, sedentary behavior, school-based intervention, ideation metatheory, qualitative study

## Abstract

**Background:**

Guided by Kincaid’s ideation metatheory, the Promoting hEalthy Diet and Active Lifestyle (PEDAL), a school-based intervention, was developed. The central tenets of ideation consist of three psychosocial domains (cognitive, emotional, and social) that are assumed to influence behavior cumulatively. This study explored the implementation of two components of PEDAL and how ideation factors influenced diet and active lifestyle behaviors.

**Methods:**

Following the conduct of two out of four intervention components (a series of interactive in-class lessons and actionable home activities), six focus group discussions were conducted with students (*n* = 16) and teachers (*n* = 7). Audio-recorded data were extracted as expanded notes and analyzed using the applied thematic analysis, with theme generation framed by the ideation metatheory.

**Results:**

While students enjoyed the lessons and home activities, they hoped that lessons could be more physically active. Teachers found lessons easy to implement but struggled with time constraints. Students reported gaining a better understanding of sedentary behaviors and habit formation through the lessons. However, they faced barriers, such as COVID-19 movement restrictions and the lack of autonomy at home, which impeded their opportunities to engage in more physical activity and healthier eating. Regarding ideation, students discussed cognitive factors that motivated behavior change, such as attitudes toward healthier behaviors and image concerns. Emotional factors, including the enjoyment of activities, influenced students’ motivation to engage in healthy eating and physical activity. Some students also reported resistance to further changes when they felt they were already “doing too much.” Social factors, such as family involvement, were critical but often limited.

**Conclusion:**

The findings have illuminated several crucial aspects of enhancing intervention implementation, supported the use of the ideation metatheory to underpin the PEDAL intervention, and guided the subsequent evaluation of the whole intervention. This study was retrospectively registered with the ISRCTN registry [ISRCTN16114046].

## 1 Introduction

Previous research has established the importance of healthy eating and physical activity in treating and preventing childhood obesity (1), and guidelines for children have been established worldwide (2, 3). However, globally, many children do not meet the guidelines for dietary and movement behavior (comprising physical activity, sedentary behavior, and sleep), contributing to the increase in childhood overweight and obesity (1). Setting the stage for healthy lifestyle behaviors during childhood, particularly adolescence (4), is crucial to avoid persistent vulnerability to unhealthy diet and movement behaviors (5). Much effort and resources have been dedicated to school-based health promotion interventions, which have been lauded as an effective approach, as they provide concentrated and continual access to children (1, 6, 7).

To maximize the potential efficacy of school-based interventions, the use of social and behavior change theories is often advocated to guide the design and evaluation of these interventions (8, 9). Using theory facilitates the understanding of behavior change and informs practitioners of the “active ingredients” and strategies that can bring about behavior change (10). Having theory components clearly stated and measured helps researchers to gauge *if* change has been achieved and *how*, such that the theory itself can be assessed and updated in tandem (10). Despite the value of using theories to underpin interventions, recent literature reviews examining childhood obesity prevention interventions found that over half of the included studies did not specify the use of theory in the interventions and that there is varied evidence on the effectiveness of interventions underpinned by theory (6, 7, 9, 11).

The mixed findings on the effectiveness of theory-based interventions could be attributed to several reasons. Firstly, it could be due to the underreporting and lack of analysis of implementation fidelity – the extent of the “active ingredients” being delivered by the implementor (12, 13). In the case of school-based interventions, challenges such as resources, teachers’ attitudes, curriculum demand, and implementation support may affect the implementation of evidence-based interventions into routine practice in schools (7). However, the lack of such information makes it difficult to make direct inferences between intervention effectiveness and the underpinning theory (13). This highlights the importance of examining the implementation of interventions to inform the design and procedures of interventions for use in real-world settings (14).

Secondly, the “light” use of theory in several studies (6, 7), where theoretical frameworks were only stated, used as background understanding, or deconstructed to examine only a few theoretical constructs rather than as a whole (15). This lack of theoretical use in this field highlights the need for the explicit use and comprehensive analysis of theory in problem formulation, guiding intervention development, and evaluation.

Lastly, the choice of theory may not have been appropriate. For example, using a theory with a focus on beliefs for a behavior that is heavily influenced by emotions (16). Systematic reviews revealed that the obesity prevention interventions for children and adolescents predominantly used the Social Cognitive Theory (SCT) and, in some cases, a combination with other theories such as the Ecological Model and Theory of Planned Behavior (TPB) (7, 9). However, evidence on the efficacy of interventions based on SCT was found to be weak (11). This suggests that interventions relying on a single theory may be insufficient to capture the complexity of lifestyle behaviors among children and adolescents, such as the interacting individual, social, and contextual influences on their behaviors (17–19), and the need to explore a more comprehensive theory or model.

One such theory is the ideation metatheory, which posits that successful interaction between resources, environment, and psychosocial factors (ideational factors) would lead to behavior change (20, 21). It draws on known constructs from multiple behavioral theories, such as the SCT, TPB, Health Belief Model, and Social Ecological Model, allowing a more comprehensive understanding of behaviors (20, 21). The ideational factors are grouped into three domains (cognitive, emotional, and social), which can be influenced by social and behavior change strategies and techniques, knowledge and skills, and the environmental context, and are assumed to function independently or synergistically to influence behavior (20, 21). These constructs in the metatheory correspond with local literature, indicating that children’s and adolescents’ health behaviors are affected by multiple levels simultaneously (22, 23). While this theory has been used to predict and influence a range of behaviors in adults, for example, breastfeeding behaviors (24) and insecticide-treated bed net use among caregivers of children (25), it has yet to be used for lifestyle interventions in children in Singapore.

This study aims to explore the implementation of a multicomponent school-based lifestyle intervention for primary school children and to unpack the influences of behavior change among children through leveraging ideation. An exploration of diet and movement ideation, specifically on healthy eating, physical activity and sedentary behaviors, among children in Singapore is also warranted to inform the evaluation of the intervention (i.e., key ideational factors to be examined as mediating variables (26)). Furthermore, assessing the feasibility of implementation approaches in early pre-testing and trialing stages of intervention will aid in understanding how best to operationalize the intervention implementation and evaluation as plans unfold (26). To enable examining both these aspects of theory-based intervention design, our research is anchored around the pre-testing of two components: (A) a series of interactive in-class health education lessons and (B) actionable home activities to support habit formation of the planned intervention, namely the Promoting hEalthy Diet and Active Lifestyle (PEDAL) program. The specific objectives are to:

1. Explore the strengths and weaknesses of implementing PEDAL Components A and B.
2. Explore the skills and knowledge transfer, environmental context, and ideational factors (cognitive, emotional, and social) influencing children’s diet and active lifestyle behaviors.

The components of the proposed ideation metatheory are detailed as the analysis unfolds.

## 2 Methods

### 2.1 PEDAL intervention

**Figure 1:**
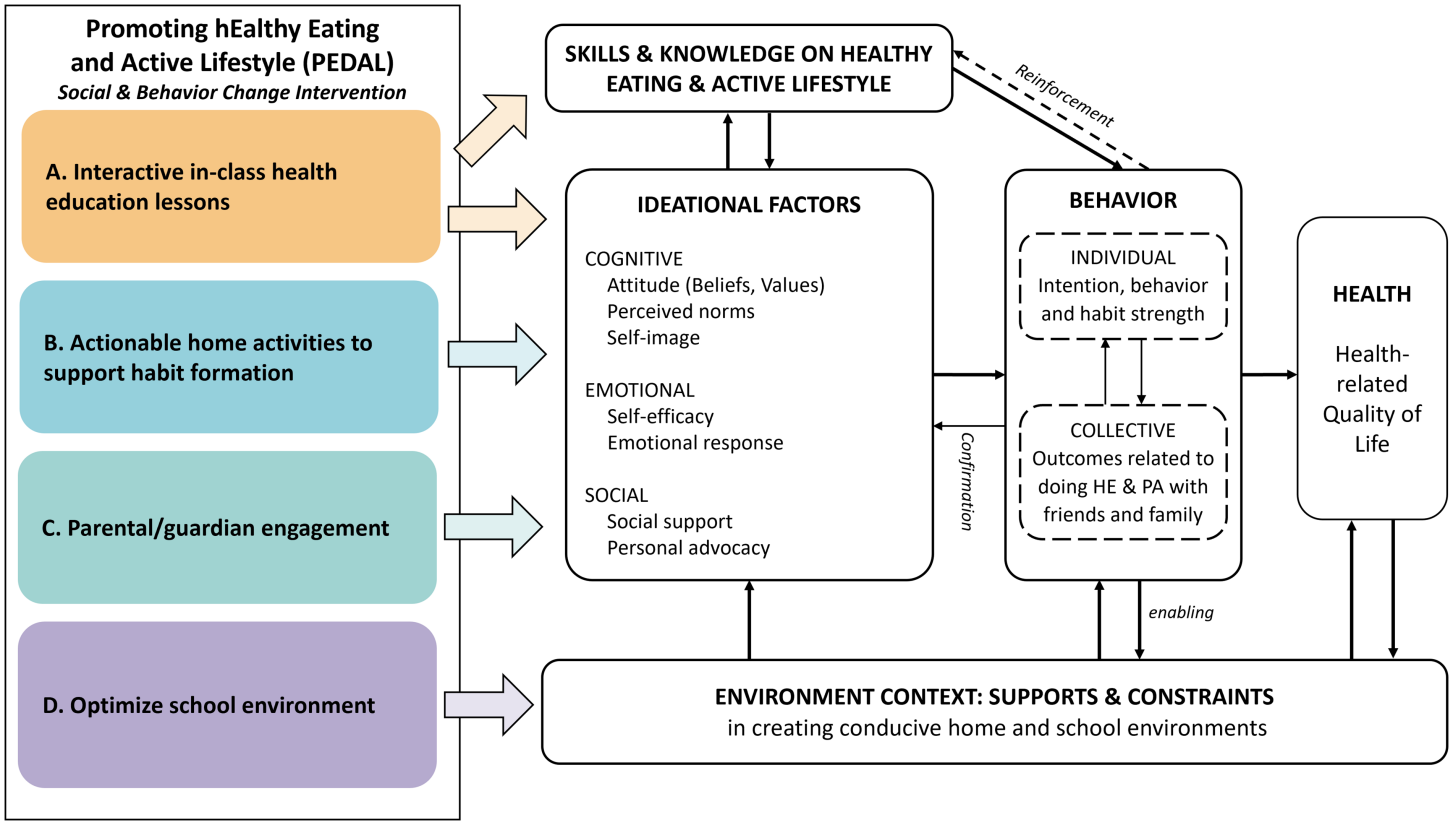
Illustration of the programme theory for the PEDAL intervention (27).

Promoting hEalthy Diet and Active Lifestyle (PEDAL) was developed as a multicomponent school-based intervention, guided by the ideation metatheory (20) (Figure 1). The full intervention protocol can be found elsewhere (27). In brief, PEDAL aims to increase fruit and vegetable intake, increase time spent on moderate-vigorous physical activity (MVPA) and decrease sedentary time in school children aged 10–11 years. PEDAL consists of four components: (A) interactive in-class health education lessons on healthy eating, sedentary behaviors, and habit formation, (B) actionable home activities to support habit formation, (C) parental/guardian engagement, and (D) optimizing the school canteen environment.

By convenience, two co-education public primary schools were recruited for this study. Physical education (PE) teachers at both schools received materials and training from the research team for Components A and B of PEDAL. Component A consists of five 30-min interactive health education lessons delivered by trained PE teachers. During each lesson, students will go through a short educational video or didactic teaching on healthy eating, sedentary behaviors, or habit formation, followed by an active in-class quiz to reinforce the concept taught. Component B involves take-home activities related to the topics in Component A, populated into a card (also known as a PEDAL card), which students were introduced to during a lesson and encouraged to complete in the subsequent lessons.

The intervention components were conducted to the Primary 5 cohort (typically children aged 10–11 years old) from January to March 2022 in the first school (referred henceforth to as S1) and from July to October 2022 in the second School (S2). During the implementation in S1, movement restrictions due to the COVID pandemic were still in force (e.g., suspension of activities during recess and after school activities). These restrictions were then relaxed during the latter half of the year when the study was conducted in S2.

### 2.2 Qualitative approach and reflexivity

A narrative qualitative study was conducted to explore students’ and teachers’ experiences after the intervention. Focus group discussions (FGDs) were conducted with students and teachers, separately in both schools. The Standards for Reporting Qualitative Research (SRQR) was used to guide the reporting of the study (23).

The study was designed by a post-graduate student (CMJC) with a background in nutrition and public health, a senior researcher with expertise in maternal and child health (MFFC), and a senior qualitative and mixed methods researcher (ZJH). Data were collected, extracted, and analyzed by CMJC with guidance from ZJH. All team members were female and had no prior relationship with the participants. The researchers’ backgrounds in child nutrition, public health, and qualitative research informed the interpretation of data, but may posed potential biases toward perspectives of behavioral change. To mitigate this, reflexive journaling, peer debriefing, and iterative team discussions were used to critically examine assumptions and ensure interpretations remained grounded in participants’ voices.

### 2.3 Participants and data collection

A total of six FGDs, three in each school, composed of two groups with students and one with teachers, were conducted. Each student focus group consisted of a mix of boys and girls from the same class, to ensure gender diversity, who volunteered to participate. A purposive sampling strategy was employed to select students based on class progress status, specifically targeting those with high and middle progress, as these students were anticipated to provide richer data relevant to the study aims. Each teacher focus group consisted of PE teachers who delivered the lessons to the students.

Semi-structured discussion guides were developed (Supplementary Marterial), principally guided by the ideation metatheory (17), to facilitate the FGDs for students and teachers. The FGDs were conducted in person, facilitated by the first author and study data analyst, CMJC, and supported by a note-taker. Before the FGDs started, students and teachers were briefed about the session, icebreakers were used to build rapport, and participants were reminded that their responses would be anonymised. All FGDs were conducted in a private location and were audio recorded. The FGDs lasted between 45 min and an hour.

### 2.4 Data analysis

After the FGDs were conducted, the data were extracted using the scribing approach (28, 29). This entailed listening and summarizing the recorded data in detail and then extracting salient verbatim quotes in tandem to support the summaries. Member-checking of extracted data was not conducted due to the young age of the student participants and the time constraints of teachers’ schedules. Extracted data were imported into Atlas.ti for coding. Applied thematic analysis (30) was undertaken, and analysis was guided by our theoretical position (31). Data were first segmented according to the research questions, before coding based on the structure and content (e.g., strengths, challenges, and ideational influences). A codebook was iteratively developed and refined to ensure consistency in coding. Themes were generated and refined through iterative comparison and discussion of codes between CMJC and ZJH. Major themes are highlighted in ***italicized bold***, and their supporting sub-themes are narrated in turn; all findings are summarized in tabular form with illustrative quotes, see Tables 1–7. Participants were assigned pseudonyms to reference quotes meaningfully while preserving anonymity.

**Table 1:** Illustrative quotes for intervention strengths from the students’ and teachers’ perspectives.

Themes	Quotes organized according to sub-theme
Genuinely engaged by most of the in-class lesson content.	In-class activities got students moving and doing things other than didactic learning. *“It was fun because usually in the class we do not really get to walk around or do any activities. We just do the Health Education book. The games were actually fun, really fun.” “We get to exercise a little bit in class, like warm up our body.” (FG5, S2 students Beth & Betsy, F)*
Lessons were easy to implement	Materials were provided and were more straightforward than the typical health education lesson plan *“I think it was quite straightforward, especially the PowerPoint slides are all already there, we just need to go through them, so it was quite easy.” (FG3, S1 teacher Audrey, F)* *“We are usually given a Health Education book to go through it. The lesson usually requires us to come up with our own content or go through the answers in the book. So, it can be quite dry. When the kids know they have to stay in class for health education, they usually feel quite disappointed because they want to go out to play.” (FG6, S2 teacher Blake, M)*
Liking the content and delivery of the intervention	Materials, games and discussions that informed knowledge transferred well *“Some of the activities from this booklet can help us be healthy, because some people in our class do not really eat healthily so this booklet guide them (to) a healthier lifestyle.” (FG5, S2 student Billy, M)* *“I think there was a session on (overcoming) barriers, which resonated with everyone. Because we planned, but most of the time, we will face difficulties and not follow up on it. So that lesson was something I will not forget. We (teachers) also got a better understanding of why students are not very excited or find it very difficult to finish the activities.” (FG6, S2 teacher Blake, M)*

**Table 2:** Illustrative quotes for intervention challenges from the students’ and teachers’ perspectives.

Themes	Quotes organized according to sub-theme
Students struggled to engage with take-home activities	Instructions were not clear to them; Period to complete the activities conflicted with examination preparation *“We do not know how to do and, especially, ‘cause we had our exams, some of us have tuition (extra classes) every day in school.” (FG1, S1 student Amelia, F)* *“I think almost none (from my class) did the take-home activities. I kept reminding them, but I think most of them did not really do that. Then I ask them how come, and they told me that they have other things to do, they have homework, and other things, so the take-home activities were like the bottom of their priority.” (FG3, S1 teacher Arthur, M)*
Teachers felt that PEDAL activities needed better alignment with the school schedule and student abilities	The shortening of activities or extending the intervention period was required *“Because when we have to get them to sit down in the classroom, it means that we probably have to manage their behavior a bit more because they are restless. Especially those that goes longer than the half an hour that we usually give for health education, when they start to fidget then it becomes a bit harder and challenging to teach.” (FG6, S2 teacher Brooke, M)* Specialized adaptation was needed so that lower-ability students might be enabled to keep up *“I think for the tail-end classes, probably if you simplify it will be better, I mean make it easier for them and not too many things for them to fill in. So, I think that will help.” (FG3, S1 teacher Arthur, M)* *“I think because it’s (the lesson content) so in-depth, and then because it was in-depth plus many questions, it became overwhelming. But if you just maybe have fewer questions, I think it will help.” (FG3, S1 teacher Audrey, F)*
Lessons can be even more active	Conducting lessons out of classroom *“Maybe after some time of sitting during the PEDAL lesson, we can play games in the PEDAL lesson.” “Maybe, play games outside the classroom.” (FG4, S2 students Bobby, M & Bella, F)* *“Because my class likes to go out for PE, so when I say we need to stay in and do some health education, like some in-class activity, then they will feel ‘sian’ (disappointed).” (FG6, S2 teacher Brooke, M)*
Implementation fidelity was high in S2, but not S1	When all lessons were not delivered, this was due to time constraints or teacher’s misunderstanding *“I did not manage to finish the Habit Formation lesson. So, the last part, where the pupils have to do the worksheet, I did not do it with them. But I did show them the video… I do not have enough time; we wanted to go through the slides and videos, but by the time we finish, it’s almost half an hour. Sometimes we do not even get to finish the whole lesson (within the scheduled time),” (FG3, S1 teacher Afiq, M)* If the content was interrupted or deemed contentious, this led to issues with fidelity *“During the video, when it’s getting to the best part, our PE teacher will pause and then talk, talk, talk.” “Ya, we get frustrated. It’s a lot to take in. Just one at a time, it’s either he talks, or the video.” (FG1, S1 students Anna & Amelia, F)* *“Our teacher says that some stuff are nearly impossible, because nobody, except for our teacher himself measures their food and try to achieve energy balance.” (FG1, S1 student Aaron, M)*

**Table 3:** Illustrative quotes for students’ acquisition of skills and knowledge from students’ perspective.

Themes	Quotes organized according to sub-themes
Gaining a better understanding of key concepts through different learning methods	Through lessons that required them to “do” rather than “listen” *“We remember this (lesson on sedentary behaviors) so much because we had to stand up and then have to show actions!” (FG2, S1 student Aiden, M)* Through examples of how to apply the concepts instead of the overarching themes *“We need to buy more groceries, that are healthy. Like if you want to buy some biscuits, try to check the ingredients. Just try to minimize those sugar biscuits, that the outer covering is sugar.” (FG4, S2 student Beatrice, F)* *“There is a lot of stuff to do, too much stuff, like English, Math, Chinese.” “There are a lot of boring stuff to remember, plus other classes.” (FG1, S1 students Alicia, F & Amos, M)*

**Table 4:** Illustrative quotes for environmental context (from school to home) from students’ and teachers’ perspectives.

Themes	Quotes organized according to sub-themes
Engagement in active activities was somewhat limited	By COVID-19 restrictions in one school *“We need to do the activity itself, to form the habit… but I cannot go outside for active lifestyle, due to COVID.” (FG1, S1 student Aaron, M)* *“We have also been restricted by the things we have to do in our school, and not allowing people to move around did not help much. We were in the space when students could not do a lot of the collective activities.” (FG3, S1 teacher Andrew, M)* Lack of time to do physical activities *“I said I will jog daily, which I do not do because Mondays I have after school things, Tuesdays I have tuition, Wednesdays I am free, but I have to do my tuition homework, Thursdays I have CCA, Fridays I have tuition, then Saturday I have tuition. So it’s like very rare for me to go out.” (FG2, S1 student Aiden, M)*
Ensuring the right resources are present	To harness current and future motivations *“In our school, during ‘Rise and shine’, we play the activities like badminton, every morning when we come to school.” “When we come to school very early, we sometimes just go and play.” (FG5, S2 students Betsy, F & Bradley, M)* *“I think the canteen food needs to get better. I think the idea of healthy eating to the kids now is that food is gonna taste bland and lousy, to be honest. So, I think it’s how to build up intrinsic motivation in the children. If healthy food tastes good, then when they grow up, they will look for something healthy. But if healthy food tastes like what they taste in the canteen, then the kids will still go back to fast foods because that honestly will taste better.” (FG6, S2 teacher Blake, M)*
The home environment was often described as restrictive	Difficult for children to find support for the PEDAL card activities *“I will be like, finally they allow me to do this!” “But I do not think my parents will do it.” (FG1, S1 students Amos, M & Anna, F)* *“We have tried to engage our parents as well but the respond from the parents were not very good. If we can overcome that challenge, I think we can win the pupils over. The stumbling block is always the parents.” (FG3, S1 teacher Afiq, M)* Difficult for children to exert autonomy over their choices *“Nah, they will not let me decide, they will cook whatever and then I’ll eat.” (FG4, S2 student Bella, F)* *“My father threatens me to eat (fruits and vegetables). He threatens me saying, ‘If you do not eat, I will not buy you a gift’.” (FG2, S1 student Aiden, M)*

**Table 5:** Illustrative quotes of cognitive factors influencing children’s behaviors from students’ and teachers’ perspectives.

Themes	Quotes organized according to sub-themes
Positive attitudes on benefiting from healthy eating and physical activity	Keeping in good shape and being able to focus schoolwork *“It’s because we need to eat healthy, to be fit and healthy.” “So, we will be healthier and we can also live longer.” (FG4, S2 students Beatrice & Bella, F)* *“It can help us be more focused on our academic subjects and less on our health because we already got it handled when we eat healthily. We do not need to spend so much time to think about our health if we are able to form the habits of eating healthily.” (FG1, S1 student Aaron, M)* Help them work toward their goals *“During lunch, I will ask for more veggies, so I can run faster.” (FG4, S2 student Bella, F)*
Keep a decent weight, look good or “get skinny”	To feel better *“Healthy and I’ll feel like I’m fit, and good when I lose weight.” (FG4, S2 student Bobby, M)* *“You can keep yourself healthy, and then you will not feel like you hate yourself.” (FG2, S1 student Aiden, M)* And fit the ‘societal norms’ of not looking too heavy *“I would feel much better, fitness wise. I will feel that I’m not so fat, not so chubby.” (FG2, S1 student Alex, M)* *“(Losing weight will) make our parents proud. Because my mother say that I am getting heavier, because I always eat a lot.” (FG4, S2 student Beatrice, F)*

**Table 6:** Illustrative quotes of emotional factors influencing children’s behaviors from students’ and teachers’ perspectives.

Themes	Quotes organized according to sub-themes
Students became emotionally engaged when activities became fun and rewarding	Using entertaining ways to persuade to engage in healthy eating and physical activities and incentivized by prizes *“The PEDAL card activities were fun, as at home I do not really do much, so this one really helped me to get active.” (FG5, S2 student Betsy, F)* *“I think if there were like something to be given to us, like prizes or something, then our parents would definitely ask us to do it.” (FG1, S1 student Amy, F)* Enjoying doing the activities with parents *“The take-home activities are very interesting. A variety of activities you can do, especially during the March holidays and (we have) nothing to do. Especially the ones that we can do with our parents, the ones that require parent’s signature. (FG2, S2 student Alex, M)* Having lost weight, and wanting to continue the habit *“Eating healthy and being more active really helped a lot. I lost a lot of weight by doing this.” (FG1, S1 student Amos, M)* *“Last time my weight was getting heavier because I was not exercising at all. I got COVID for 7 to 8 days, so I did not go outside, so I did not get to exercise, so that’s why.” (FG4, S2 student Bobby, M)*
Students and teachers expressed their doubts on whether students will apply what they have learned	Despite understanding the importance of healthy eating and physical activity *“Yup, they do see an importance, but whether they live it out is a different story.” (FG6, S2 teacher Blake, M)* Due to the complexity and efforts involved *“It’s very troublesome, like, you cannot possibly measure your food every single day. Whatever you drink is whatever you drink. You cannot possibly, like, go back into the trash, take the can out to check for the nutrition information.” (FG1, S1 student Anna, F)* *“It’s actually very hard to, because we already form this kind of ‘bad’ habit, it’s very hard to follow this and change our habits.” (FG1, S1 student Aaron, M)*
Boomerang effect	Doing too much makes them want to do less, those who think they are already healthy/active will not do more *“My family will (eat healthy and engage in physical activities) with me but sometimes I will feel like this is too much, can we just take a break. I’ll be the one who is saying no, and it must be moderated. ‘Cause if you do too much, obviously you will not like it anymore.” (FG1, S1 student Amelia, F)* *“My physical activity level before and after (the intervention) is the same; I have 5 h of exercise every week. [On changes in diet] (I’m) not sure… I’m naturally quite a healthy person.” (FG2, S1 student Alex, M)*

**Table 7:** Illustrative quotes of social factors influencing children’s behaviors from students’ and teachers’ perspectives.

Themes	Quotes organized according to sub-themes
Challenging to get parents and siblings involved in the intervention	Lack of time from parents; parents not prioritizing non-academic-related work *“My parents have no time because all of them are working. They will be like, go online and learn stuff, why did not you do this assignment, huh, huh!” (FG1, S1 student Aaron, M)* *“When I go home and tell my parents (about PEDAL), they will say ‘Ok, Ok’ but they do not actually listen.” (FG4, S2 student Beatrice, F)* Lack of engagement from siblings *“When I ask my sister to help me (with the home activities), she will be like, ‘No, I want to sleep, bye.” (FG1, S1 student Anna, F)*
Socializing with peers and peer rivalry was shown to help motivate engagement with activities	Making activities social *“I hate going out alone whenever I’m going to exercise. So, I will call my friend, he’s my neighbor, so I’ll call him and he will say ‘Ok, let us go’. Then we both go out, and then we will stay out for 4 to 3 h. We’ll go cycling or we will go for a walk, and we like to hang out a lot.” (FG2, S1 student Aiden, M)* *“I want to start a club about healthy eating, with my friends. We’ll eat healthy every day, bring our water bottle filled with plain water, and we will jog around the school once.” (FG4, S2 student Beatrice, F)* Adding competitive elements to activities *“The only thing we talk about (to our friends) is ‘how many PEDAL stars you have?’. Everyone likes to compare… We are very competitive. We want to be the best of the best.” (FG2, S1 student Alex, M)*

To ensure trustworthiness and rigor of the qualitative analysis, several strategies were employed. Credibility was enhanced through triangulation of teacher and student responses, enabling cross-validation of findings across different participant perspectives. Dependability was ensured via iterative refinement of the codebook and repeated discussions between CMJC and ZJH to ensure consistency and reliability in interpretation. Confirmability was supported through reflexive team meetings during which assumptions and potential biases were examined to keep the analysis grounded in participants’ voices. Transferability was facilitated by providing descriptions of the participants’ characteristics and the school context, enabling readers to assess the applicability of the findings to other settings. Thematic saturation was monitored throughout data collection through reflective discussions following each FGD. By the second FGD in each school, no new themes were identified, indicating that saturation had been achieved.

## 3 Results

A total of *n* = 23 students (*n* = 16, aged 10–11 years old), and teachers (*n* = 7) participated in the FGDs. There were equal numbers of boys (*n* = 4) and girls (*n* = 4) from each school. All PE teachers (*n* = 5, *n* = 1 female) involved in S1 participated, while only two of the four PE teachers (both male) in S2 participated in the FGD.

### 3.1 Objective 1: implementation of components A and B of the pedal intervention

#### 3.1.1 Strengths

Most students expressed that they were ***genuinely engaged by most of the in-class lesson content*** (Table 1). This was because in-class activities got students moving and doing things other than didactic learning. Teachers felt that ***lessons were easy to implement*** (Table 1), especially as materials were provided and were more straightforward than the typical health education lesson plan, for which they usually had to source their materials. Teachers also shared that the content of the PEDAL lessons made health education less boring than the typical health education lessons.

Most teachers and a few students expressed ***liking the content and delivery of the curriculum*** (Table 1). They felt that the materials, games, and discussions that informed knowledge transferred well to the students. Some teachers also highlighted that the curriculum taught students how to apply the knowledge learned, making it more relevant and easier to remember.

#### 3.1.2 Challenges

On the other hand, participation in take-home activities appeared to be limited as ***students struggled to engage with these*** (Table 2). The students who participated reported enjoying the experience, while those who did not participate reported that the instructions were unclear to them and the period to complete the activities conflicted with examination preparation. When students have to decide between working on the take-home activities or examination preparation, the latter takes priority. Other reasons included a lack of family support, discussed in the ideational sections below.

For teachers, the timings of in-class activities were raised, and it was stressed that the ***health curriculum needed better alignment with the school schedule and student abilities*** (Table 2). For example, the time allocated for health education lessons is typically 30 min, but in practice, they had less than 30 min, as time is needed to manage students’ behavior; adding topics also added to the teaching burden. Thus, shortening activities or extending the intervention period was required. This, and specialized adaptation, was needed so that lower-ability students might be enabled to keep up.

Teachers reported that lessons engaged students, but the structure seemed repetitive and predictable, making students less engaged near the end of the intervention. Although lessons were less sedentary, some students and teachers explained that ***lessons could be even more physically active*** (Table 2), e.g., conducting lessons outside the classroom.

With regards to fidelity, ***implementation fidelity was high in S2, but not in S1*** (Table 2), as not all educational lessons and home activities were conducted in S1, but all were implemented in S2. When all lessons were not delivered, this was due to time constraints or the teacher’s misunderstanding. For instance, one teacher misunderstood that certain lessons were linked and skipped one of the lessons when he could not complete the previous lesson. Also, if the content was interrupted or deemed contentious, this led to issues with fidelity.

### 3.2 Objective 2: exploring the skills and knowledge, environmental context and ideational factors influencing healthy eating and active lifestyle behaviors among children

#### 3.2.1 Skills and knowledge for healthy eating and physical activity

Students reported **gaining a better understanding of key concepts** (e.g., sedentary behaviors, forming habits) **through different learning methods**, differentiated by students’ ability levels and room for simplifying some materials as previously identified (Table 3). Students were able to better recall lessons that required them to “do” the activities rather than “listen” during the lessons, and examples of how to apply the concepts, instead of the overarching theme. This could be because students connected more positively to the active in-class games, which are the “fun” or less “boring” aspects of the intervention, as reported in the previous section. One student also explained that they could not clearly remember the overarching theme, which was perceived as uninteresting and technical, like the other academic topics they were expected to remember.

#### 3.2.2 Environmental context: support and constraints

***Engagement in active activities was somewhat limited*** by COVID-19 restrictions in S1 as movement restrictions were enforced during the study period (Table 4). This limited students’ opportunity to participate in active activities as face-to-face interaction with other students in the school was limited, and they were not allowed to participate in outdoor play. Besides this, students also reported that they lack time to participate in physical activities due to the significant amount of academic-related work they have to fulfil.

Students in S2 had more opportunities to be active in school as restriction measures were lifted during the study period. Despite having more opportunities, ***ensuring the right resources are present*** is crucial in harnessing current and future motivation (Table 4). For example, having enough sports equipment (e.g., racquets, balls) available for more students during unstructured play time in school and improving the quality of food provided in the school canteen. The school environment should, therefore, be equipped to make healthy experiences accessible and memorable, as good experiences with healthy choices reinforce the pleasure students gain, encouraging them to repeat these experiences in the future.

In terms of home environment, ***it was often described as restrictive*** as parental control over students’ decisions was shown to be very strong (e.g., making decisions about meals, restricting time spent on non-academic related work, or being coercive about eating healthy) (Table 4). Students also find it challenging to garner family support to do the PEDAL card activities at home, despite many sharing that they enjoyed involving their parents and would have liked to have them involved. Family support is further unpacked in the ideational sections below. At the same time, it was also difficult for children to exert autonomy over their choices, thus making them feel that they do not have control over their present and future decisions.

#### 3.2.3 Ideational factors – cognitive factors

***Positive attitudes towards healthy eating and physical activity*** were shared, such as the benefits of keeping in good shape and being able to focus on schoolwork (Table 5). Some students also view healthy behaviors as beneficial if the behaviors help them work toward their goals, such as having better athletic performance or being a health-conscious chef in the future. Although these benefits were valued, they were not always seen as important as learning and doing well in core academic subjects. Students also discussed image-driven motivations; these include ***keeping a decent weight, looking good or “getting skinny,”*** to feel better and to fit the ‘societal norms’ of not looking too heavy (Table 5). These responses suggest that while students were cognitively aware of the health benefits of healthy eating and physical activity, their motivations to engage in these behaviors were often intertwined with their self-image and perceived norms.

#### 3.2.4 Ideational factors – emotional factors

***Students became more engaged when activities were fun and rewarding***, for example, using entertaining ways to educate and motivate them to engage in healthy eating and reduce sedentary time, being incentivized by prizes, or simply doing the activities with their parents (Table 6). Students also find it rewarding if they have had prior positive experiences from engaging in healthy behaviors (e.g., having lost weight and wanting to keep that going). On the other hand, the issue of self-efficacy was raised, where some ***students and teachers expressed their doubts on whether students will apply what they have learned*** in their lives due to the complexity and efforts involved, despite understanding the importance of healthy eating and physical activity (Table 6). A ***boomerang effect*** was elicited from students’ sharing – some of them mentioned that “doing too much” of certain behavior (e.g., physical activity) can be off-putting and would opt for moderation of their current behaviors (Table 6). Also, those who regarded themselves as ‘quite healthy’ did not feel the need to engage in more healthful behaviors (e.g., doing more sports or eating more healthily).

These responses highlight the role of positive emotional responses, where enjoyment and positive affect strengthen motivation and behavioral intention. They also reveal that nuances of self-perceptions around health status can both reinforce and hinder sustained engagement in healthy behaviors among children. Moreover, the findings suggest that cognitive awareness alone may not translate into action when confidence in one’s ability to maintain behavior is low, underscoring the importance of self-efficacy in mediating the link between knowledge and practice.

#### 3.2.5 Ideational factors – social factors

It was evident that whole family involvement was pivotal to participating in home activities and making lasting changes, and poignantly, as mentioned earlier, students really wanted the interaction with their families. However, it was ***very challenging to get parents and siblings involved*** in the take-home activities; often, the reason was a lack of time from parents, parents not prioritizing non-academic related work and the lack of engagement from siblings (Table 7). ***Socializing with peers and peer rivalry were shown to help motivate engagement with activities*** by making activities social or adding competitive elements to activities (Table 7).

This highlights the centrality of social support from family and peers in influencing behavior change. While the lack of active engagement from familial networks can limit the development of supportive norms around healthy living at home, peer interactions serve as a key social catalyst. Specifically, encouragement and competition among peers, reflecting social influence, help normalize healthy practices and strengthen behavioral intentions.

## 4 Discussion

Overall, the findings highlighted aspects that were done well and some challenges faced while implementing the interactive in-class health education lessons (Component A) and actionable home activities (Component B) of the PEDAL intervention. The exploration of ideation on diet and active lifestyle behaviors in this study provided valuable insights into how knowledge, environment, and ideational factors can influence behavior change in healthy eating and physical activity among primary school children in Singapore.

From the discussions with students and teachers, it was evident that lessons that were active and perceived as fun made learning and teaching healthy behaviors more enjoyable. This aligns with previous research indicating that higher perceived enjoyment facilitates engagement and positive attitudes toward participating in physical activity and healthy eating among children (32–34). Our findings also showed that less sedentary lessons helped students remember better, supporting the theory of embodied learning, where bodily movements enhance learning in children (35). These insights suggest that curriculum design for health education should focus not only on the content but also on how positive emotional responses can be elicited through interactive, playful, experiential learning approaches.

Besides making lessons fun, our findings underscored the importance of flexibility in implementation, while also recognizing the need for a balance between flexibility and fidelity. Granting teachers the autonomy to adapt the implementation process allows the intervention to better meet the unique needs and dynamics of individual schools and classrooms. For instance, teachers may divide a single lesson into two if more time is needed to provide additional explanations or examples of taught topics. This capacity for adaptation has been shown to enhance teacher willingness to deliver the intervention, thereby facilitating successful implementation (36, 37). However, a careful balance must be struck between flexibility and fidelity in implementation (38). While flexibility allowed teachers to tailor the intervention to their specific classroom context, it also revealed potential pitfalls. For example, one teacher’s disagreement with the lesson content negatively influenced students’ attitudes. This outcome likely arose from the limited opportunities for teachers to voice their concerns and dissent with the research team, which could have been mitigated by allocating more time for rapport-building between the research team and teachers early in the process. This experience highlights the importance of prioritizing teachers’ perspectives during training sessions, in addition to familiarizing teachers with the materials and intervention expectations (38). From a policy and curriculum design perspective, these findings highlight the need to equip teachers not only with content knowledge but also with structured platforms to voice concerns, clarify doubts, and share implementation experiences.

The exploration of diet and active lifestyle ideation in this study identified several ideational factors that could influence behavior change among children. Under cognitive factors, these include attitudes toward healthy eating and physical activity (e.g., knowing the benefits motivates students to change their behaviors), and self-image (e.g., self-perception of “already healthy” demotivates them from doing more). Emotional factors include emotional responses (e.g., positive experience motivates engagement) and self-efficacy (e.g., high complexity and effort reduce motivation to engage). Social factors, such as social support from family and friends, were also mentioned. Additionally, having the appropriate knowledge and supportive environmental conditions facilitated positive behavior changes in children. These findings align with past studies that examined the key determinants influencing children’s and adolescents’ eating and physical activity habits in the Western (39, 40) and local context (22, 23), supporting the inclusion of these factors in PEDAL’s program theory. While the identified ideational factors broadly align with existing literature, our findings indicate that emotional factors, particularly enjoyment and self-efficacy, were especially salient in shaping children’s motivation and engagement. This suggests that interventions aiming to promote healthy behaviors in children may benefit from placing greater emphasis on eliciting positive emotional experiences and strengthening confidence to act on acquired knowledge.

Among the ideational factors, it was unexpected that a theme on image-driven motivation was elicited among children in this age group, as this was more often observed among older adolescents between 12 and 18 years old (41). A possible reason for such early image-driven motivation could be the early exposure to social media (41), suggesting that the social media environment and exposure could be a potential factor to explore among children in this age group.

Also, it was interesting that a “boomerang effect,” unintended negative intervention effects being reverberated instead of intended positive effects, was observed. This phenomenon can be explained using the theory of psychological reactance, which posits that individuals react negatively if they perceive that their behavioral freedoms are being threatened (42). Our findings exemplified this concept, revealing that some students, particularly those who perceived their current diet and activity levels to be sufficiently healthy, were put off when asked to do more physical activity and eat more fruit and vegetables. Thus, careful planning of the design and implementation of intervention messages to address potential misperceptions and provide appropriate encouragement without triggering reactance is warranted to prevent such unintended consequences. Enhancing students’ self-efficacy could also help motivate children to sustain their healthy eating (43) and physical activity (44) behaviors. For example, learning how to cope with challenges faced when engaging in positive behaviors (45).

While many of the ideational factors tended to be at the personal level, our findings alluded that factors from the social and physical environment were also crucial in influencing behavior change and habit formation among students. Past studies have found that it is often the social support from family and friends and the accessibility of a supportive environment that encourages children to take action, improving their self-efficacy (39, 46). Moreover, the perception of satisfaction obtained from performing healthy behaviors with family and friends and the availability and accessibility of healthy foods and play areas, as shown in this study, are essential for initiating and maintaining behaviors, which is also the starting point for habit formation (47, 48). This, thus, highlights the importance of intervening in children’s social and physical environments to improve their dietary and active lifestyle behaviors (49, 50). Identifying ideational factors in this study will help guide the operationalization and evaluation of trialing the full PEDAL intervention (26). The implementation of intervention components A and B, designed to address the identified ideational factors, was well-received by both the students and teachers and has been shown to motivate some students to initiate positive behavior changes. Furthermore, the findings underscored the importance of extending the intervention beyond student education to encompass both the home and school environments, supporting our plans for components C and D (parental engagement and optimizing school environment) of the PEDAL intervention.

To our knowledge, this is the first study in Singapore to explore the implementation and the role of ideation in behavioral change to inform the development of a tailored intervention. However, a few limitations were identified for this study. The students’ perspectives from this study may not be generalizable to all primary school children across Singapore, as the study was only conducted among the Primary 5 students in two schools. Students from other levels or schools, particularly private schools, may have different views on the intervention implementation and different ideational influences. Thus, further study is warranted to explore the views of other schools prior to implementing similar obesity prevention interventions. During the FGDs, socially desirable responses may also have been elicited. Teachers were aware that the facilitator was part of the research team that developed the intervention, and students may have perceived the facilitator as a teacher figure, potentially limiting the extent of critical feedback provided. Although responses from teachers and students were compared to enhance credibility, the triangulation in this study was limited to qualitative sources. Incorporating additional data points, such as quantitative measures of behavioral outcomes or classroom observations, could provide stronger methodological triangulation and validation of self-reported perceptions. Moreover, the current study design precluded assessment of longer-term or sustained behavioral changes, and a longitudinal follow-up would be valuable to evaluate whether short-term ideational shifts translate into lasting health behaviors.

## 5 Conclusion

The findings of this study have shed light on several crucial aspects of improving intervention implementation, such as making intervention activities more enjoyable, allowing flexibility and adaptation, and addressing teachers’ concerns before intervention delivery. Findings also supported the utilization of the ideation metatheory as an underpinning framework, as well as the plans for parental engagement and the optimization of the school environment components of the PEDAL intervention. These insights inform the refinement and evaluation plans for a future pilot study of the PEDAL intervention and serve as a valuable resource for future intervention research in this area of public health.

## Data Availability

The raw data supporting the conclusions of this article will be made available by the authors, without undue reservation.
