# Supplementary material for: Exploring the implementation and theory of a school-based diet and active lifestyle intervention among primary school children
Source: Front Public Health. 2025 Dec 15;13:1708767. doi: 10.3389/fpubh.2025.1708767 (PMC12745290; doi:10.3389/fpubh.2025.1708767)
Supplement: Supplementary file 1 [file Table_1.DOCX]

## Appendix. Topic guides used for the FGDs

**Students’ Group Discussion Topic guide**

**Icebreaker:** What is the first word that comes to your mind when you think of the school’s Health Education lesson?

***Objective 1: Student’s extent of awareness of PEDAL lessons regarding knowledge/skills retention***

- What do you think the main purpose of the PEDAL lesson was?
- What are some key messages you can remember from each lesson?
- Why do these messages stick in your head? What is important about them?
- Have you discussed about these issues with your parents? What did you talk about?

***Objective 2: Feedback on PEDAL lesson, materials and ideational factors***

*On lessons and materials*

- Who thought the lessons were great? What did you really like about these?
- Who thought they were just OK? Why?
- How about the PEDAL card? How did you find the instructions and activities?

*Ideational factors*

- Do you think the people around you (friends and family) are eating healthily and doing physical activities? What about yourself, do you think you are someone who live healthily?
- How do you think you can make eating healthy and being active more fun or easy to do?
- What kind of rewards do you think you and your classmates would like to have after completing the cards?
- Can you share any times when you have discussed about the things you learnt in the lessons and activities at home or outside of class with friends, what kinds of things to you all talk about?

***Objective 3: Student’s perspectives on behaviour change and habit formation***

- Is there any diet or physical activity change that you have made after going through the PEDAL lessons?
- Do you think you will continue doing the things you have learnt? Why/ why not?
- If you do eat healthier and increase PA, how does that make you feel about yourself? Why?

***Close out:*** Is there anything else you would like to tell us before we close the session?

**Teachers’ Group Discussion Topic guide**

**Icebreaker:** What is the first word that comes to your mind when you think of the school’s Health Education lesson?

***Objective 1: Teachers’ views on how engaging the PEDAL lessons are***

- What are your main concerns about PEDAL achieving it’s intended goal, i.e., of helping children on a trajectory to better lifestyle choices and wellbeing?
- What are some key messages that you felt stood out most from each lesson?
- Why do these messages stick in your head? What is important about them?
- What do you think about parental engagement in PEDAL, how do you see this side of things working, or not working out?

***Objective 2: Teacher’s feedback on the feasibility of PEDAL implementation, views on lesson materials and how the programme might be better related to ideational factors***

*On lessons and materials*

- How enjoyable did you find facilitating the lessons? Why?
- What do you think about the PEDAL lesson plans and materials?
  - Is the content pitched at a level suitable for P5 students?
  - Is there anything you would like to change about the lessons and/or the materials provided?
- Did you manage to implement all of the activities as they were designed? Why/why not?
  - Have you done anything differently from the teacher’s guide that worked well?
  - What helped or hindered the implementation of the activities?
- How about the PEDAL card? Was it useful, clear, and pitched at the right level for helping students build healthy habits? What do you think are some reasons why students complete/ did not complete the activities?
- Do you think PEDAL is feasible to implement as part of the health education curriculum?

*Ideational factors*

- How can we enhance the feeling of being rewarded for children when they complete PEDAL tasks or follow healthy eating and physical activity habits?
- What do you think motivates participation, and specifically makes activities more fun? Or increases their confidence, making healthy habits feel easy to do?
- How do you think the school can help to promote consistent norms around healthy eating and physical activity?

***Objective 2: Teachers’ perspectives on behaviour change and habit formation***

- Will you continue to apply the information you have learned through PEDAL in your teaching? If so, how or if not, why not?
- Do you see the students making any diet or physical activity changes after going through the PEDAL lessons?

***Close out by asking them to each say what they liked most about the discussion and end the session.***
